# Concurrent psychiatry for patients enrolled in opioid agonist treatment: a propensity score matched cohort study in Ontario Canada

**DOI:** 10.1186/s13011-019-0213-6

**Published:** 2019-06-26

**Authors:** Kristen A. Morin, Joseph K. Eibl, Joseph M. Caswell, Graham Gauthier, Brian Rush, Christopher Mushquash, Nancy E. Lightfoot, David C. Marsh

**Affiliations:** 1 Laurentian University, Sudbury, Canada; 2 Northern Ontario School of Medicine, Sudbury, ON P3E 2C6 Canada; 3 Canadian Addiction Treatment Centres, Richmond Hill, ON Canada; 4 Centre for Addiction and Mental Health, Toronto, Canada; 5 Department of Psychology, Lakehead University, Thunder Bay, Canada; 6 Institute of clinical and Evaluative Sciences, Sudbury, ON Canada

**Keywords:** Opioid use disorder, Mental disorders, Mental Health services, Psychiatry, Concurrent Health services, Rural Health

## Abstract

**Objective:**

The objective was to characterize the relationship between geography, concurrent psychiatric services, all-cause mortality, and acute health care use for individuals enrolled in Opioid Agonist Treatment, in Ontario, Canada.

**Methods:**

We conducted a propensity score matching study of patients enrolled in Opioid Agonist Treatment in Ontario for the first time between January 1, 2011, and December 31, 2015. We first compared outcomes between patients who were actively engaged and patients who were not actively engaged in Opioid Agonist Treatment. We created treatment and a control groups on the basis of an individual’s access to psychiatric care within an episode of Opioid Agonist Treatment. Relative risk and number needed to treat were calculated to determine the correlation between psychiatric care and health outcomes among patients enrolled in Opioid Agonist Treatment at two time points within an episode of care and for two geographic regions in Ontario (north and south).

**Results:**

During the first year of Opioid Agonist Treatment, concurrent psychiatric care was associated with a reduction in all-cause mortality in southern Ontario (RR 0.80, 95% CI, 0.73–0.87), a reduction in emergency department visits in both northern and southern Ontario (north: RR = 0.76, 95% CI, 0.72–0.81; south: RR = 0.87, 95% CI, 0.86–0.88), and a reduction in hospitalizations (north: RR = 0.88, 95% CI. 0.82–0.94, south: RR = 0.92, 95% CI, 0.91–0.93).

**Conclusion:**

Our findings have significant clinical and political implications for health system planning highlighting the need for integrated mental health and addiction services for individuals with Opioid Use Disorder.

**Electronic supplementary material:**

The online version of this article (10.1186/s13011-019-0213-6) contains supplementary material, which is available to authorized users.

## Background

Opioid use disorder (OUD) is a significant and growing contributor to the global burden of disease [1] and opioid poisoning has been characterized as a crisis devastating families and communities [2] across Canada. In 2016, there were nearly 3000 opioid-related deaths in Canada [3] which accounted for nearly 30,000 years of life lost [4]. It is estimated that 50 to 90% of patients with OUD have a concurrent mental disorder and that patients with this type of co-morbidity have a significant increased risk of death, infectious disease, acute and chronic health complications [5–8]. Despite these factors, there are challenges with coordinating mental health and addiction services, and connecting people to the appropriate clinical services based on their needs withing the current health system in Ontario [9].

The opioid crisis in northern Ontario communities is severe, and is especially dire in rural and remote areas [10]. Northern communities experience some of the highest rates of opioid prescribing and opioid-related deaths in the province [11]. For example, a community in the northwest region of the province had the second highest opioid-related death rate (9.3 per 100,000 population) and two other communities in the northeast region, also ranked in the top 10 communities with relation to opioid-related death rates (11.9 per 100,000 population) between 2004 and 2006. This compared to the provincial rate of 4.7 per 100,000 population during the same time period [11]. Remote communities often face barriers when accessing various forms of health services, including addiction and mental health services [12–14]. Barriers can include having to travel long distances to access care, geographic isolation, and a general lack of health human resources [12, 15]. For example, a report by Health Quality Ontario found that in 2009, the Toronto Central region had eight times more psychiatrists per 100,000 population than the northern regions (62.7 in Toronto central vs. 8.3 and 7.1 per 100,000 in the north east and north west Ontario) [16].

Many strategies to address the opioid crisis have focused on reducing exposure to opioids and monitoring physician prescribing practices, as well as enhancing access to opioid agonist treatment (OAT) [17]. OAT is currently the standard of care and the intervention with the best evidence for long term patient safety, social wellness, and physical health benefits for the treatment of OUD [18]. For instance, in a recent meta-analysis based on 16 studies, Ma, J. et alfound that untreated participants had higher all-cause mortality (RR = 2.56,95% CI,1.72–3.80) and overdose mortality (RR = 8.10, 95% CI, 4.48–14.66]) when compared to those actively engaged in OAT [19]. In Ontario, patients receiving OAT will frequently commence treatment at a specialized addiction clinic [20]. These specialized clinics are generally funded by the provincial physician fee-for-service compensation model [21], and often operate separate from other health and social services [22].

A key element of OAT treatment, according to Health Quality Ontario’s Opioid Use Disorder Quality Standards, is that patients receive integrated, concurrent, and culturally safe management of their physical and mental health, as well as additional addiction treatment and social needs [23, 24]. However, clinics, especially those located in rural communities, have limited ability to provide all-encompassing care within the current model of care in Ontario, due to the resource intensive nature and cost of comprehensive OAT services combined with lack of coordination across disparate funding programs [22].

Decades of evidence have established the complexity of substance use disorders and our understanding of how positive treatment often come as a result of a combination of medical, psychological, social and environmental interventions [25–27]. The literature demonstrating the impact of psychiatric interventions offered in conjunction with medications for the treatment of OUD is conflicting. Studies have demonstrated that mental health services improve the clinical course for OAT, but that benefits are dependent on factors such as the program characteristics and type of therapy [25]. Other studies have found that mental health services have no effect on treatment outcomes for patients enrolled in OAT [25, 28].

Despite the conflicting literature, federal and provincial government bodies in Canada have highlighted the high health, social, and economic burden of comorbidities of mental and substance use disorders [7, 29–32], and have recommended the integration of mental health and addiction services [33–35]. To provide the highest quality of care to this unique and vulnerable population, further research must be conducted to better understand implications of concurrent services for patients enrolled in OAT in Ontario. In this study, we investigated the relationship between concurrent psychiatric care, all-cause mortality, and acute health service use including emergency department (ED) visits and hospitalizations in northern and southern regions of Ontario.

## Methods

### Data

The study was approved by the Research Ethics Board of Laurentian University in Sudbury. Data were obtained by submitting a formal requisition to ICES. ICES, formally known as I*nstitute for Clinical Evaluative Sciences, is an independent, non-profit entity which* compiles provincial data on administrative and health indicators which can be linked across various health care settings The data were accessed remotely using a secure server located at ICES. We used ICES administrative data from January 1, 2011 through to December 31, 2016. ICES provided anonymized individual-level inpatient data collected from Ontario publically funded health services. The datasets consisted of patient demographic information including: age, sex, place of residence, neighborhood income quintile and mortality from the Registered Persons Database (RPDB), a record for each inpatient discharge from all publically funded Ontario hospitals from the Discharge Abstract Database (DAD), a record for each out-patient use of all publically funded Ontario emergency departments (ED) from the National Ambulatory Care Database (NACRS), a record of each patient publically funded drug prescription from the Ontario Drug Benefit Plan Database (ODB), a record of each patient encounter with a physician (including any diagnosis given by the physician) from the Ontario Health Insurance Plan Database (OHIP). Patient information was linked anonymously across databases using encrypted 10-digit health card numbers. The linking protocol has been described extensively elsewhere [36, 37], and is used routinely for health system research in Ontario [38–40]. Data sources are described in detail in Additional file 1.

### Patient groups

#### Primary cohort

**Fig. 1:**
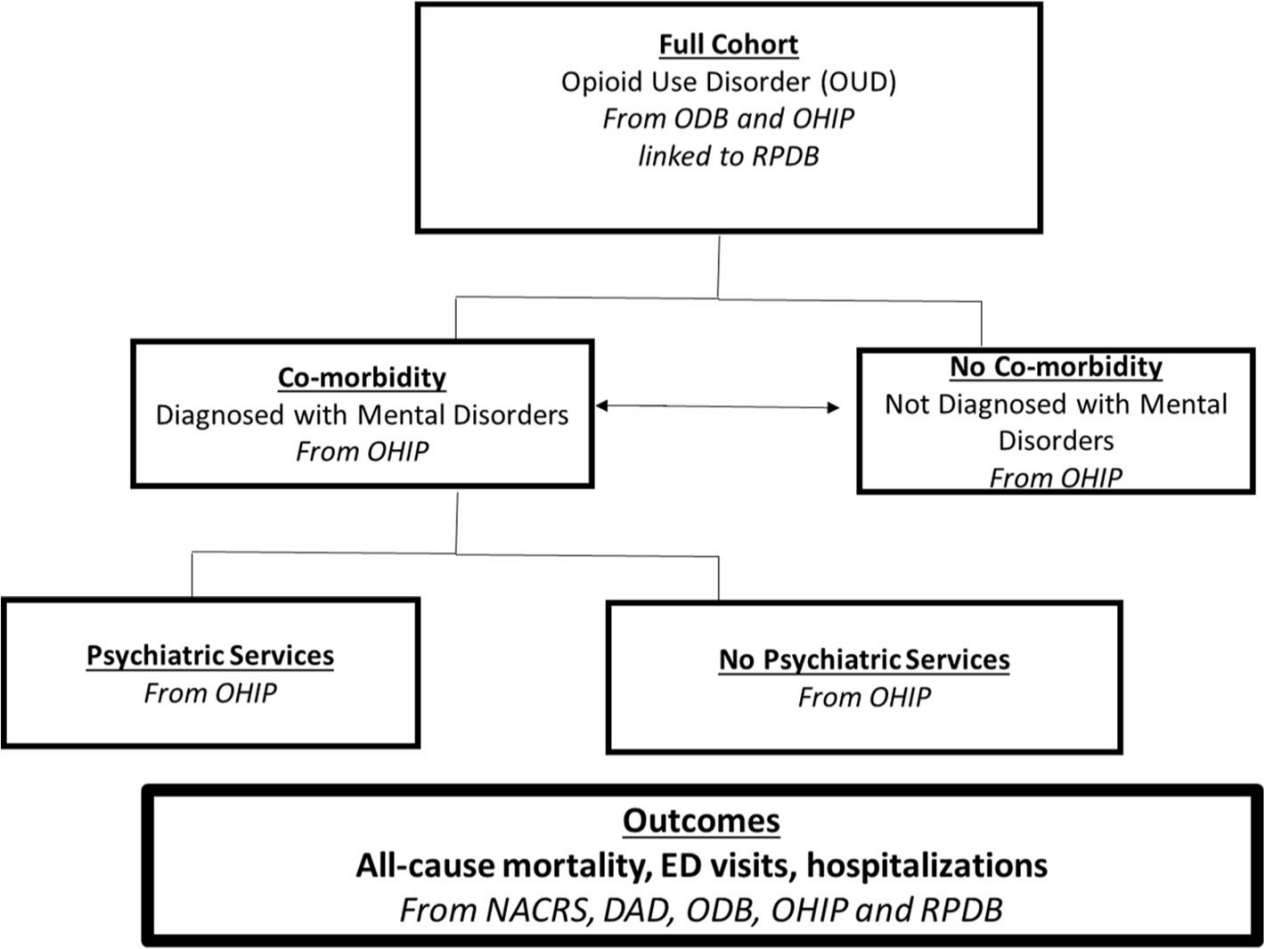
Patient Groups and Data Sources

Patients were included in study if they had the following characteristics: 1) their first OAT visits between January 1, 2011, and December 31, including methadone and buprenorphine/naloxone, 2) they were 15 years or older. Access to OAT has been used as a proxy to identify the prevalence of Opioid Use Disorder (OUD) [11, 41, 42]. Both OHIP and ODB databases were used to identify patients. The total number patients in the cohort was 55,921 (the number patients identified first from OHIP billing was 40,398, the number of patients first identified from ODB was 13,314, and the number of patients who had been identified both in OHIP and ODB was 2209). Patients were excluded based on the following criteria: 1) if they were prescribed methadone in a tablet formulation greater than 20% of their methadone prescriptions over a one-year period,, 2) if there was missing patient information regarding place of residence, age, or sex. In Ontario, methadone used for the treatment of OUD is dispensed exclusively in liquid formulation and these patients were likely being treated primarily for chronic pain (Fig. 1). All patients were followed from their date of first OAT initiation to the date of treatment discontinuation with one-year follow-up, or end of the study period (December 31, 2016).

#### Mental disorders group

Patients were assigned to only one of the following groups: Diagnosed with a mental disorder and not diagnosed with a mental disorder. To be assigned to mental disorders group, an individual must have had an ICD-9 or ICD-10 diagnosis, in any diagnostic position in the medical record, indicating a mental disorder (Additional file 2). For example, individuals assigned to the mental disorders group would have a mental disorder-related ICD-9 or ICD-10 diagnostic codes in any of their inpatient records (in any diagnostic position) from one-year prior to the time of their first OAT event to one-year after the date or their last OAT event (or the study end date). Studies indicate that if there is a mental disorder diagnosis in administrative data sets, there is a strong likelihood that the data capture is accurate [43]. However, if a mental diagnosis does not appear in the physician billing codes, the individual may still have a mental disorder diagnosis [43]. An important limitation to mention related to this variable is that we only considered people who had accessed treatment, however, the number of people with mental disorders who had not sought out treatment for remains unknown [44]. Additionally due to the nature of the data, we were not able to consider patient history or whether the disorder was an acute or chronic condition. Importantly, this variable will allow us to broadly explore how mental disorders and OUD impact the Ontario population.

#### Concurrent psychiatry services group

Patients were assigned to one of the following groups: received mental health services from a psychiatrist and not received mental health services from a psychiatrist. To be assigned psychiatric care group, an individual must have had an outpatient mental health service encounter from a psychiatrist identified by OHIP billing, in any position in the medical record (Additional file 3). For example, any individual assigned to the psychiatric care group would have a mental disorder-related ICD-9 or ICD-10 diagnostic codes and have received outpatient mental health from a psychiatrist 30 days prior, during or 30 days after being actively receiving OAT services. It is important to note that we were limited to only examine OHIP-billed mental health services, which by default excluded the consideration of community mental health services and federally-funded health services. This includes omission of federally-funded mental health services provided in Indigenous communities, as well as any mental health services funded by a provincial ministry other than the Ontario Ministry of Health and Long Term Care. Additionally, due to the nature of the data, we were not able to consider any historical of mental health services within our analysis. It is important to note that the intensity of the psychiatric care (i.e. number of visits) was not measure for this study due to the fact that the average number of psychiatry visits per year was 1.2 and patients were only for 1 year of active OAT.

### Actively engaged in OAT

All patients were followed after their first treatment episode, to a maximum follow-up date of December 31, 2016. Continuous OAT was assessed on the basis of prescription refill or physician encounter within 30 days of the event (i.e. no gap in receiving medication of greater than 30 days). 30 days was chosen based on the use of this interval in previous research [32–34]. The database used for medication dispensing in this study might not capture doses administered in hospital or provincial correctional settings. However, in Ontario, patients will typically continue to receive methadone or buprenorphine in these settings. Since most hospital admissions or provincial incarcerations are less than 30 days, this approach allows the analysis to be conducted without misinterpreting such events as treatment interruption.

### Baseline variables

#### Location of residence

Patient postal codes were used to determine their location of residence at the outset of OAT. Provincially defined health regions (Local Health Integration Networks) [45] were used to stratify patient location of residence into northern and southern regions. Local Health Integration Networks (LHINs) are regional health authorities who govern the administration of health service funding across defined geographic areas of Ontario. LHIN 13 and 14 were used to describe northern Ontario and the remainder were used to identify southern Ontario regions. The Statistics Canada Rural and Small Town definition was used to distinguish between rural and urban areas [46]. Next, we created geographic groups defined as: northern rural, northern urban, southern rural and southern urban. Location of Residence was considered a baseline covariate. Therefore, it was only considered at the index date and we did not account for the fact that patient can move between rural and urban areas within the analysis.

#### Income quintile

Income quintile is a variable ascertained using neighborhood-level metrics (postal codes) from Census data. Therefore, it is important to note that information can be misclassified if a low income family was living in a neighborhood classified as high income or vice versa. Income quintile was considered a baseline covariate. Therefore it was only considered at the index date and we did not account for patients moving between neighborhoods.

#### Human immunodeficiency virus (HIV)

HIV positive patients were derived from the OHIP database. ICES uses an algorithm that identifies an individual with HIV when there have been three physician claims in 3 years. HIV can be an indication of the complexity of patients with Opioid Use Disorder. HIV was considered a baseline covariate.Therefore HIV status was considered as positive only when identified at the index date.

#### Deep tissue infection

Deep Tissue Infections Deep were derived from the OHIP database. Three distinct deep tissue infections were included: septic arthritis, osteomyelitis, and infective endocarditis. Deep tissue infections are pathogenic infections involving subcutaneous tissues that may proliferate to surrounding tissues and muscles [47]. This variable was included particularly because this type of infection is related to the introduction of bacteria into the venous circulation or subcutaneous which is common among those who inject drugs [48, 49]. Deep tissue infections can also be an indication of the complexity of patients with Opioid Use Disorder. Deep tissue infection was considered a baseline covariate, therefore, is were identified at the index date for this study.

### Outcome measures

#### All-cause mortality

One of the main outcome variables for this thesis was all-cause mortality (ACM). ACM was not limited to opioid overdose deaths, but includes opioid-related and non-opioid related mortality data. Other studies have had difficulties ascertaining the nature of the fatalities occurring among people who use opioids [50]. For instance, the percentages of overdose deaths classified as undetermined vary greatly in the United States, ranging from 1 to 85% between 2008 and 2010 [51]. At the time of the study, mortality specific data was not available therefore, we chose to examine deaths from any cause as an outcome within our cohort. The ACM variable allowed us to avoid omitting deaths from causes other than opioid-overdose that are important to consider for this population such as suicide and accidents. Many reports highlight strong correlations with the increasing availability of prescription opioids [52–55] as the main cause of rising mortality rates.

#### ED visits and hospitalizations

Both ED and hospitalizations were linked to the encrypted patient identifier as well as to the index date, which is defined as the first enrollment in OAT. ED visits and hospitalizations were studied as binary variables. ED were captured as ‘frequent ED visits’ if a patient had a count of over 10 ED events in a publically funded Ontario hospital within a 1 year. The count of ED events in this population was exceptionally high in our cohort (i.e. mean of 15.9 events per year). We based our decision to dichotomize ED visits (> = 10 and < 10) based on studies which tried to define frequent use of emergency departments [56–59]. In the literature, the definition of a frequent user has been based upon the number of attendances within a given time frame ranging from 3 to 12 attendances within a year [56]. Hospitalizations were captured if a patient had one hospital admissions in a publically funded Ontario hospital within 1 year. The count of hospitalizations in our cohort was much lower than ED events (i.e. mean of 4.1 events per year). ED and hospitalizations were captured in three groups: opioid-related, mental health-related, and for reasons other than mental health or opioids. Both frequent ED visits and hospitalization are metrics which are used by health system planners and funders in Ontario to understand gaps of services in communities [58, 60, 61]. From this lens, frequent events in acute care settings, will be interpreted as negative health outcomes in this study.

#### Matching

Propensity scores were calculated using logistic regression to balance important baseline covariates between treatment groups in each strata. Predicted probabilities were logit transformed before matching. We used a greedy matching algorithm and cases were matched on a caliper width of 0.20 times the standard deviation of the logit propensity scores. We used greedy matching to produce matched samples with balanced covariates across the treatment and control group. The algorithm generates a one-to-one matched pairs. The algorithm will choose the participant with the highest propensity score in the treatment group and match a control group member with the closest propensity score. It will then choose a second treatment group member with the next highest propensity score and match the second treatment participant. The algorithm repeats the process until all participants are matched [62]. Balance of covariates, which included age, sex, income, rurality, HIV status, and deep tissue infections were assessed (before and after matching) using standardized differences (d) where d ≥ 10% indicates a clinically-relevant difference.

### Statistical analysis

We first calculated relative risk (RR) to determine the correlation between OAT treatment and the outcomes of interest including: all-cause mortality, ED visits and hospitalizations in order to determine the baseline rates for the study cohort. We did this by comparing patients in OAT against patients outside an OAT episode of care. Next, we used McNemar’s test [63] to determine statistical significance of the associations between concurrent psychiatric services and the outcomes of interest within an OAT episode of care only. Clinical descriptors were calculated including RR, and number needed to treat or number needed to harm (NNT/NNH), along with 95% confidence intervals (CI) for each descriptor calculated using methods appropriate for dependent samples. Clinical descriptors were calculated at two time points within the OAT episode of care (at 3 months and at 12 months) for both northern and southern Ontario strata. All statistical analysis was conducted using SAS Version 9.4 [64].

## Results

### Cohort characteristics

Our cohort was composed of 48,935 individuals who had been enrolled in OAT at least one time and received a diagnosis of another mental disorder during the study period between 2011 and 2016. We stratified the cohort into two groups based on their place of residence (northern Ontario (*n* = 7233, 14.7%) and southern Ontario (*n* = 41,702, 85.2%)). Of the patients with OUD, in northern Ontario 4.55% (*n* = 329) received concurrent psychiatric care within the first 3 months of their OAT episode of care.In southern Ontario 15.51% (*n* = 6467) received concurrent care. Among the 7233 patients with OUD in northern Ontario, 1, 470 (20.3%) were retained for at least 1 year in OAT. In southern Ontario 9, 500 (22.8%) were retained for at least 1 year in OAT. For those retained for 1 year in OAT, 11.28% (*n* = 816) received concurrent psychiatric care at some time during the first 12 months of OAT in northern Ontario and 26.58% (*n* = 11,083) in southern Ontario. We calculated standardized differences (d) to highlight differences between the treatment (those with OUD and a mental disorder who received concurrent psychiatric care) and control groups (those with OUD and a mental disorder who did not receive concurrent psychiatric care) before matching.

Before matching, we found that patients residing in northern Ontario were a heterogeneous group, where standardized differences indicated an imbalance between treatment groups with regards to sex (d = 17.53), age (d = 25.17), income (d = 28.61) and OAT retention (d = 13.50). In the southern Ontario cohort, standardized differences indicated a balance between the treatment and control group where there were no standardized differences over 10% in any of the covariates (Table 1). After matching, standardized differences (d) were less than 10% for all patient characteristics (Table 2), which indicates balanced covariates between the treatment and control group. Table 2 demonstrates the rigor of the matching procedure.

**Table 1:**
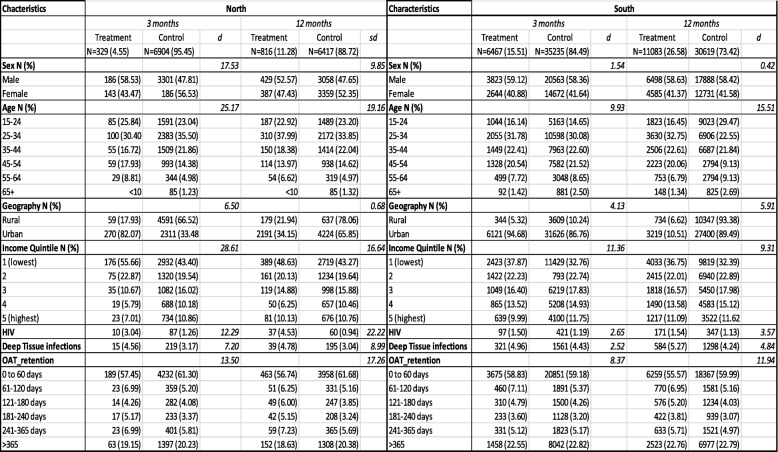
Pre-Matched Characteristics of Patient Groups

**Table 2:**
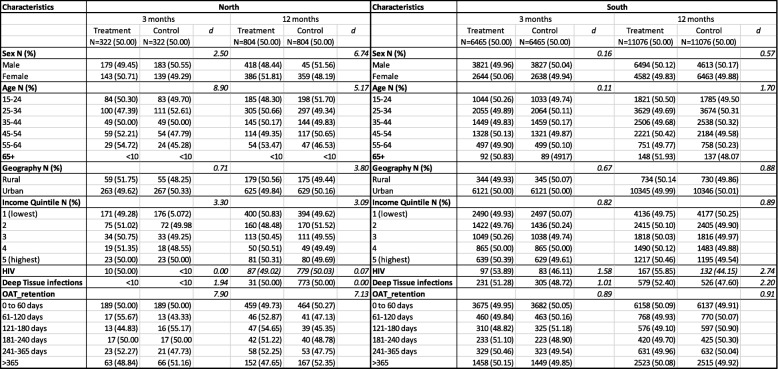
Post-Matched Characteristics of Patient Groups

### Outcomes

**Fig. 2:**
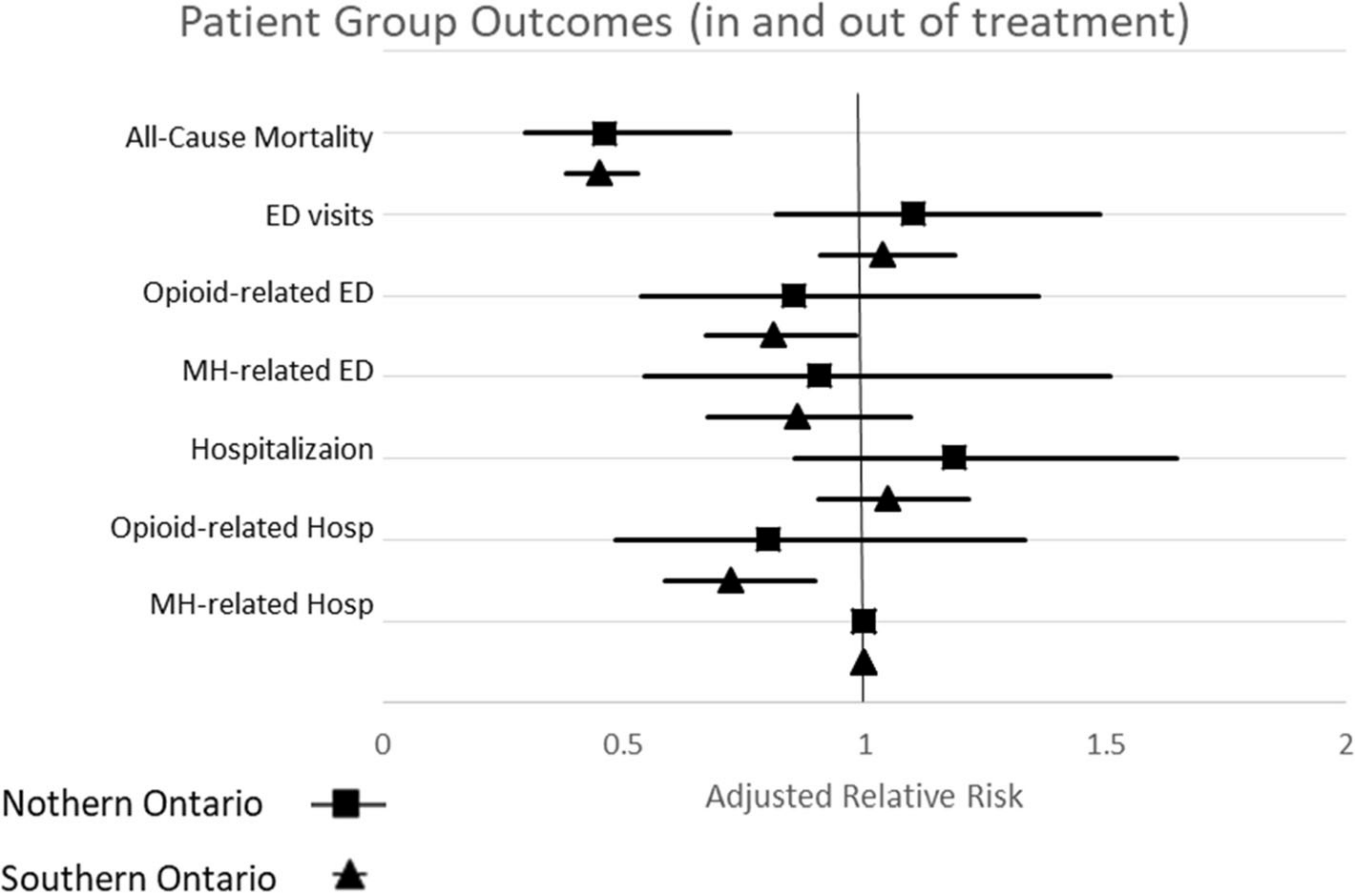
Relative Risk for In vs. Out of OAT

Using logistic regression, we calculated an adjusted relative risk (aRR) of patients diagnosed with Opioid OUD, who also received a diagnosis of another mental disorder to study outcomes including ACM, ED visits and hospitalizations for patients actively engaged in an OAT episode of care and those not actively engaged in a treatment episode. After adjusting for age, sex, rurality, income, deep tissue infections, HIV status and retention in OAT, we observed that patients actively engaged in OAT were associated with a 56% reduction in ACM when compared to patients not engaged in OAT in northern Ontario (aRR = 0.44, 95% CI, 0.38–0.52) and 55% reduction in southern Ontario compared to the time periods when they were not receiving OAT. We observed no significant impact of being enrolled in OAT on proportion with frequent ED visits for reasons other than mental health or opioids in either geographical region (north: (aRR = 1.02, 95% CI, 0.82–1.50, south: (aRR = 1.038, 95% CI, 0.91–1.88). However, there was a 19% reduction in the risk of frequent opioid-related ED visits in southern Ontario (aRR = 0.81, 95% CI, 0.67–0.98) but no significant impact of being enrolled in OAT on frequent opioid-related ED visits in northern Ontario when compared with patients not engaged in OAT (aRR = 0.85, 95% CI, 0.34–1.36). No significant impact of being enrolled in OAT was detected on frequent mental health-related ED visits (north: (aRR = 0.91, 95% CI, 0.54–1.51, south: (aRR = 0.86, 95% CI, 0.68–1.10)) when compared to the reference group. In addition, we observed that patients actively engaged in OAT had no significant association with a reduction in hospitalizations for reasons other than mental health and opioids. We observed a 28% reduction in opioid-related hospitalizations during OAT in southern Ontario (aRR = 0.72, 95% CI, 0.58–0.90), but no significant reduction in northern Ontario (aRR = 0.80, 95% CI, 0.48–1.34). Enrolled in OAT did not have a significant impact on the number of mental health-related hospitalizations. Results described in Fig. 2. Results are outlined extensively in Additional file 4.

Next, using McNemar’s test we evaluated the associations between concurrent psychiatric care while actively in treatment and ACM, frequent ED visits and hospitalizations. We tested for associations at two different time points (3 months and 12 months) within patients’ OAT episode of care. We also tested for differences by geographical regions in Ontario (northern Ontario and southern Ontario).

**Fig. 3:**
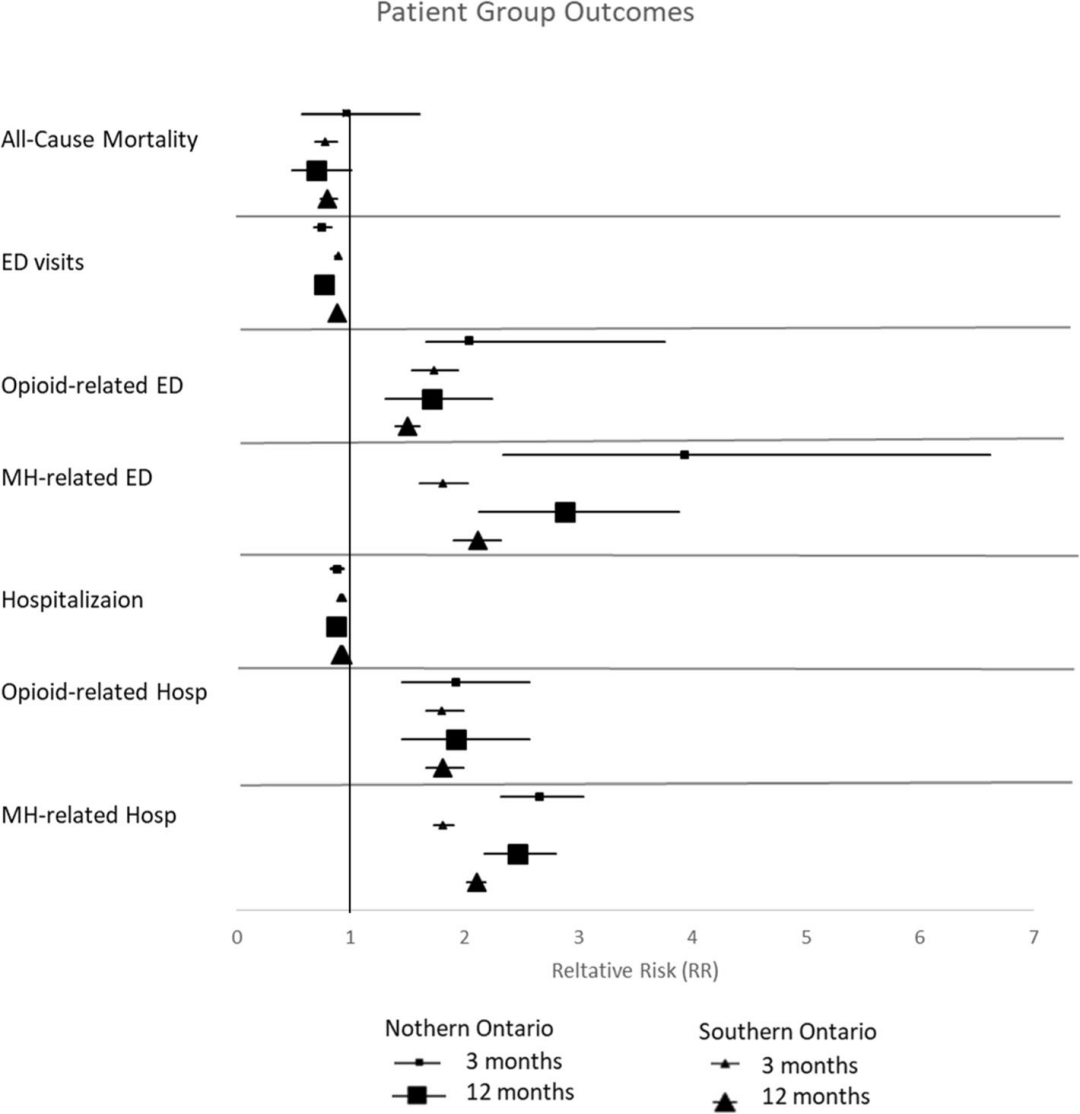
All-Cause Mortality and Acute Health Service Use During the First 3 and 12 Months of a Concurrent Episode of Care by Place of Residence

There was no statistically significant reduction in ACM from receiving concurrent psychiatric care in northern Ontario (3 months, RR = 0.79 95% CI, 0.49–1.26; 12 months, RR = 0.70, 95% CI, 0.49–1.01). In southern Ontario, concurrent psychiatric care during the first 3 months of OAT was associated with a 16% reduction in ACM (RR = 0.84 95% CI, 0.75–0.94) and concurrent care during the first 12 months of OAT was associated with a 20% reduction in ACM (RR = 0.80, 95% CI, 0.73–0.87). This indicates that 69 OAT patients with a mental disorder would need to be treated with concurrent psychiatric services during the first 3 months (NNT = 63, 95% CI, 63–29,428) or 57 OAT patients with a mental disorder (NNT = 57, 95% CI, 57–41,154) during the first 12 months of their OAT episode of care to reduce one death in the population in southern Ontario. Results described in Fig. 3 and extensively in Additional file 5.

In northern Ontario, concurrent psychiatric care for OAT patients with a mental disorder was associated with a 27% reduction in ED visits, at 3 months (RR = 0.73, 95% CI, 0.66–0.80), and a 24% reduction in ED visits at 12 months following initiation of OAT (RR = 0.76, 95% CI, 0.72–0.81). We compared this to Southern Ontario, where we observed that concurrent psychiatric care for patients with OUD and a diagnosis of a mental disorder was associated with an 11% reduction in ED visits at 3 months (RR = 0.89, 95% CI, 0.87–0.90), and a 13% reduction in ED visits at 12 months following initiation of OAT (RR = 0.87, 95% CI, 0.86–0.88). In northern Ontario NNT = 5 (95%CI, 5–1253), and in southern Ontario, NNT = 9 (95% CI, 9–17,242) for offering concurrent psychiatric care during OAT for those with OUD and a mental disorder in order to reduce the rate of frequent ED visits.

The northern Ontario treatment group had an increased risk of opioid-related ED visits of 93% at 3 months, and 175% at 12 months (3 months: RR = 1.93, 95% CI, 1.30–2.88, 12 months: RR = 2.75, 95% CI, 1.98–3.80). In comparison, the southern Ontario treatment group had an increased risk of frequent opioid-related ED visits of 43% at 3 months and 52% at 12 months (3 months: RR = 1.43, 95% CI, 1.30–1.57, 12 months: RR = 1.52, 95% CI, 1.41–1.64). The northern Ontario treatment group had an increased risk of frequent mental health-related ED visits of 294% at 3 months and 105% at 12 months (3 months: RR = 3.94, 95% CI, 2. 34–6.62, 12 months: RR = 2.26, 95% CI, 1.72–2.95). The southern Ontario proportion of frequent mental health-related ED visits was 81% at 3 months and 105% at 12 months (3 months: RR = 1.81, 95% CI, 1.61–2.03, 12 months: RR = 2.05, 95% CI, 1.87–2.24). While overall ED visits were reduced for those patients with OUD and a mental disorder who received concurrent psychiatirc care, this group of patients had higher ED visits specifically for opioid-related and mental health related problems. This suggests that the patients on OAT who received concurrent care had higher severity of mental health and substance use problems than those who did not receive cocurrent psychiatric care. Results described in Fig. 3 and extensively in Additional file 5.

In northern Ontario, concurrent psychiatric care was associated with a 12 and 13% reduction in hospitalizations at 3 and 12 months respectively (at 3 months, RR = 0.88, 95% CI, 0.82–0.94, at 12 months, RR = 0.87, 95% CI, 0.84–0.90). In southern Ontario, concurrent psychiatric care was associated with an 8% reduction in hospitaliztions, at both time points (RR = 0.92, 95% CI, 0.91–0.93). We also calculated the number needed to treat (NNT) to reduce one hospitalization in the population. In northern Ontario NNT = 8 (95% CI, 8–1828), and in southern Ontario, NNT = 14 (95% CI = 14–30,894). Results described in Fig. 3 and extensively in Additional file 5.

Patients who received concurrent psychiatric services in northern Ontario had an increased risk of opioid-related hospitalizations of 100% at 3 months and 174% at 12 months (3 months: RR = 2.00, 95% CI, 1.31–3.05, 12 months: RR = 2.74, 95% CI, 1.97–3.482). Patients who received concurrent psychiatric services in southern Ontario had an increased risk of opioid-related hospitalizations of 73% at 3 months and 87% at 12 months (3 months RR = 1.73, 95% CI, 1.54–1.94, at 12 months RR = 1.87, 95% CI, 1.70–2.05). The northern Ontario treatment group had an increased risk of mental health-related hosspitalizations of 156% at 3 months and 147% at 12 months (3 months: RR = 2.47, 95% CI, 2.18–2.80, at 12 months: RR = 2.47, 95% CI, 2.18–2.80). The southern Ontariotreatment group had an increased risk of mental health-related hospitalizations of 86% at 3 months and 108% at 12 months (3 months: RR = 1.86, 95% CI, 1.77–1.96, at 12 months: RR = 2.08, 95% CI, 2.00–2.17). Results described in Fig. 3 and extensively in Additional file 5.

## Discussion

The literature on the benefits of concurrent mental health services offered in conjunction with OAT is conflicting. Much of the literature has evaluated outcomes such as retention in treatment and mortality. Our results corroborate other studies which conclude that concurrent mental health services are predictive of better outcomes for patients enrolled in OAT [30, 65]. However, there is limited literature on ED visits and hospitalizations as negative health outcomes for this population.

In a previous study, we revealed that individuals enrolled in OAT with concurrent mental disorders had approximately twice the amount of ED visits and hospitalizations per year and had a 40% increased likelihood of ACM compared to individuals with no mental disorders. Overdose-related deaths have more than tripled between 1999 and 2016 [66]. However, there are difficulties in ascertaining the manner of the fatality [50]. For instance, percentages of overdose deaths classified as undetermined vary greatly in the United States, ranging from 1 to 85% between 2008 and 2010 [51]. Despite this, there are difficulties in ascertaining the nature of the fatalities occurring [50]. We chose to examine deaths from any cause as an outcome within our cohort, to avoid omitting deaths from causes other than opioid-overdose that are important to consider for this population such as suicide and accidents. Additionally, the data on opioid-related deaths was not available at the time of study due to delays in reporting. We found that patients who received concurrent psychiatry in southern Ontario had a significant reduction in ACM, but the correlation between psychiatric care and ACM was not statistically significant in northern Ontario. Given that the rates of ACM are already significantly reduced for patients actively engaged in OAT (54% in northern and 55% in southern Ontario), we expected the correlation to be modest for this outcome [19].

Our findings also demonstrate that access to concurrent psychiatric care may reduce the risk of frequent ED visits and hospitalizations (for reasons other than opioid and mental health) during the first year of their OAT treatment episode. Importantly, we found that one patient having repeated ED visits can be prevented by treating a moderate number of patients (NNT = 5 in northern Ontario and NNT = 9 in southern Ontario). Additionally, we observed that concurrent care seemed to have a stronger relationship with reduction in ED visits and hospitalizations in northern Ontario when compared to southern Ontario. Considering the rates of ED visits in the north are generally higher due to limited access to health care services [16], it is not surprising that when the level of care is increased, ED visits and hospitalizations are significantly reduced in northern Ontario. This finding was also interesting since when comparing patients actively engaged in OAT to those out of OAT, ED visits were not reduced in northern Ontario. Our finding therefore suggests that the additional component of concurrent care, adds a further benefit to patients engaged in OAT.

Despite the reduction of frequent ED visits and hospitalizations for reasons other than opioids or mental health, patients who received concurrent care during an OAT episode had higher opioid-related and mental health-related ED visits and hospitalizations compared to those who did not receive concurrent mental health services. The data comparing patients in and out of treatment demonstrated that OAT was correlated with a reduction in opioid-related ED visits. Ttherefore we expected that adding an additional component to existing OAT would have modest effects. This finding also speaks to the potential clinical complexity and high needs of patients who received psychiatric care and OAT. It might be that patients with OUD are self-medicating their mental disorders. Alternatively, it could be that prolonged opioid use creates chaos in individuals’ lives that later develop into mental disorders. Studies on alcohol use found this reciprocal relationship where the mental disorder predicts alcohol use and alcohol use predicts mental disorders [63, 64]. Additionally, this finding indicated that the current publicly funded system may not be meeting the needs of patients with concurrent mental disorders and OUD since concurrent care had no effect on the rate of mental health-related ED and hospitalizations.

Our study has strengths and limitations that merit discussion. The use of a large database allowed us to robustly examine the issue of OUD and psychiatric treatment on a population level over a period of 5 years. Our use of a population health approach enabled us to broadly analyze and compare a specified group of patients across Ontario as well as conduct a sophisticated analysis which replicated a randomized clinical trial with administrative data. This type of critical analysis would be very difficult to achieve if we studied a more targeted group in a smaller scale study; however, using secondary data has its limitations. Firstly, the use of physician billing is dependent on the accuracy and reliability of recording practices. Moreover, factors such as service volume, quality of care, location and coordination between physicians and organizations are missed in this type of population health approach. Additionally, we were limited to only examining OHIP billed mental health services. This by default excluded the exploration of private and community mental health services, and Federally funded health services – for instance mental health services provided in Indigenous communities – as well as any other mental health services funded by a provincial ministry other than the Ministry of Health and Long Term Care. Lastly, it is important to consider that, for those patients where mortality occurred during the first year of OAT, the likelihood of one-year retention is reduced. However, the function of time is a modest bias since we observed increase in ED visits and hospitalizations in the groups with highest mortality.

## Conclusion

The outcomes of our study have important implications for those involved in health care planning and policy development. Our data suggests that psychiatric services may help reduce the risk of mortality and reduce the use of acute care services for the most complex patients. Our findings further highlight the complexity of patients with concurrent mental health and OUD and underline gaps in treatment availability and effectiveness in northern, rural regions of Ontario. Currently, the funding and model of care for OAT in Ontario promotes access to care, but it does not incentivize efforts towards coordination with other parts of the health care system. Our study does not suggest that coordinated care should be implemented for all OAT patients. However coordinated care should be considered for those complex patients who are high users of health care services. Further research is needed to determine the volume of care needed and the effectiveness of other forms of mental health services offered concurrently with OAT services.

## Additional files

Additional file 1: Databases. (DOCX 13 kb)

Additional file 2: ICD9 and ICD10 Mental Health Diagnosis Definitions. (DOCX 32 kb)

Additional file 3: Definitions of Physician- Based Mental Health Services. (DOCX 12 kb)

Additional file 4: **Figure 2.** Supplimentary Table (Relative Risk for In vs. Out of OAT). (XLSX 11 kb)

Additional file 5: **Figure 3.** upplimentary Table (All-Cause Mortality and Acute Health Service Use During the First 3 and 12 Months of a Concurrent Episode of Care by Place of Residence). (XLSX 11 kb)

## Abbreviations

ACM: All-Cause Mortality
aRR: Adjusted Relative Risk
CI: Confidence Interval
CIHI: Canadian Institute for Health Information
d: Standardized Differences
DAD: Discharge Abstract Database
ED: Emergency Department
HIV: Human Immunodeficiency Virus
ICD: International Classification of Disease
LHIN: Local Health Integration Network
MH: Mental Health
NACRS: National Ambulatory Care Reporting System
NNH: Number Needed to Harm
NNT: Number Needed to Treat
OAT: Opioid Agonist Treatment
ODB: Ontario Drug Benefit Plan
ODPRN: Ontario Drug Policy Research Network
OHIP: Ontario Health Insurance Plan
OUD: Opioid Use Disorder
RPDB: Registered Persons Database
RR: Relative Risk
SAS: Statistical Analytics Software
