# Correction to: Concurrent psychiatry for patients enrolled in opioid agonist treatment: a propensity score matched cohort study in Ontario Canada

**DOI:** 10.1186/s13011-019-0233-2

**Published:** 2019-11-06

**Authors:** Kristen A. Morin, Joseph K. Eibl, Joseph M. Caswell, Graham Gauthier, Brian Rush, Christopher Mushquash, Nancy E. Lightfoot, David C. Marsh

**Affiliations:** 1 Laurentian University, Sudbury, Canada; 2 Northern Ontario School of Medicine, Sudbury, ON P3E 2C6 Canada; 3 Canadian Addiction Treatment Centres, Richmond Hill, ON Canada; 4 Centre for Addiction and Mental Health, Toronto, Canada; 5 Department of Psychology, Lakehead University, Thunder Bay, Canada; 6 Institute of clinical and Evaluative Sciences, Sudbury, ON Canada

**Correction to: Subst Abuse Treat Prev Policy (2019) 14: 29**

**https://doi.org/10.1186/s13011-019-0213-6**

Following publication of the original article [1], we have been notified that the following changes should occur in the content of the article. The details are below.

1. Table 1 and Table 2: the last section on OAT retention pertaining to treatment retention was only conducted on a subcohort of the population and therefore the numbers are not accurate and should be removed.
2. The article Outcome section on OAT retention should be removed because retention was not used as covariate.
