# Supplementary material for: Concurrent psychiatry for patients enrolled in opioid agonist treatment: a propensity score matched cohort study in Ontario Canada
Source: Subst Abuse Treat Prev Policy. 2019 Jun 26;14:29. doi: 10.1186/s13011-019-0213-6 (PMC6595572; doi:10.1186/s13011-019-0213-6)
Supplement: Supplementary file 1 — Databases. (DOCX 13 kb) [file 13011_2019_213_MOESM1_ESM.docx]

Additional file 1

| Data | Source | Definition |
| --- | --- | --- |
| IDENTIFIERS |  |  |
| ID | OHIP, ODB | Encrypted health card number or other identifier (based on the date of first encounter in OAT (2011-2015)) |
| Cohorts | OHIP, ODB | Mental disroders, no mental disorders |
| PATIENT CHARACTERISTICS |  |  |
| Age | OHIP, ODB | over 15 years of age (age groups in increments of 5 years) |
| Sex | OHIP, ODB | Male, Female |
| Income | RPDB | 1 (lowest), 2, 3, 4, 5 |
| Geography | RPDB | Postal Codes used to define group (northern rural, northern urban, southern rural, southern urban) |
| *North/South* |  | Geographical location: North (LHINs 13 and 14) and South (LHIN 1-12) |
| *Rural/Urban* |  | Rurality: SAC (statistical Area Classification) type: urban (Census Metropolitan Area and Census Agglomeration) and Rural (Census metropolitan influenced zones=MIZs). |
| HIV | OHIP | yes, no |
| Deep tissue infection |  | Codes: endocarditis (429), osteomyelitis (730), and pyogenic arthritis (711) (ICD9) |
| OUTCOMES |  |  |
| All Cause Mortality | RPDB | Death data based on the date of first encounter in OAT (2011-2015) |
| Emergency department visits | NACRS | Include all unplanned emergency department visits as a measure of morbidity |
| Hospitalizations | DAD | Include all hospitalizations as a measure of morbidity |
| One-year treatment retention | ODB | One-year uninterrupted treatment (2 week grace period) |
