# Supplementary material for: Concurrent psychiatry for patients enrolled in opioid agonist treatment: a propensity score matched cohort study in Ontario Canada
Source: Subst Abuse Treat Prev Policy. 2019 Jun 26;14:29. doi: 10.1186/s13011-019-0213-6 (PMC6595572; doi:10.1186/s13011-019-0213-6)
Supplement: Supplementary file 3 — Definitions of Physician- Based Mental Health Services. (DOCX 12 kb) [file 13011_2019_213_MOESM3_ESM.docx]

Additional file 3

| **Definitions of Physician Mental Health Services** | | |
| --- | --- | --- |
| **Physician model of practice** | **OHIP fee code** |  |
| **Psychiatry** | **A190** Special psychiatric consultation  **A193** Specific assessment  **A194** Partial assessment  **A195** Consultation  **A196** Repeat consultation  **A395** Limited consultation  **A695** Neurodevelopmental consultation  **A795** Geriatric psychiatric consultation  **A895** Consultation in association with special visit to a hospital in-patient, long-term care in-patient or emergency department patient  **K187** Acute post-discharge community psychiatric care, to K195, K196, K197 or K198  **K188** High risk community psychiatric care, to A190, A191, A192, A195, A197, A198, A695, A795, K195, K196, K197 or K198  **K189** Urgent community psychiatric follow-up, to A190, A195, A695 or A795  **K192** Individual hypnotherapy  **K195** Family psychotherapy - out-patients  **K197** Individual out-patient psychotherapy  **K203** 4 people group therapy  **K204** 5 people group therapy  **K205** 6 to 12 people group therapy  **K208** 2 people group therapy  **K209** 3 people group therapy  **K630** Psychiatric consultation extension  **K701** Mental health out-patient case conference |  |
